# Power and entrepreneurship

**DOI:** 10.1007/s11187-022-00660-3

**Published:** 2022-08-03

**Authors:** David B. Audretsch, Antje Fiedler

**Affiliations:** 1 Institute for Development Strategies, Indiana University, Indianapolis, IN USA; 2 Management and International Business, University of Auckland, Private Bag 92019, Auckland, New Zealand

**Keywords:** Power, Entrepreneurial opportunities, Economic sociology

## Abstract

Entrepreneurship research has benefited from embracing three economic sociology lenses—networks, cognition, and institutions—but has treated power mainly implicitly. This paper pioneers how the concept of power can advance research into entrepreneurship. We illustrate how state actors, legacy firms, and entrepreneurs variously exert coercive, persuasive, and authoritative forms of power over entrepreneurial opportunities or exercise power to pursue them as free actors. We explicitly link context and opportunity-development processes through a power lens and show how power’s interaction-focused and episodic nature that can transcend geographical and institutional boundaries might enrich entrepreneurship research.

## Abstract (plain language summary)

We argue that the field of entrepreneurship will benefit from more explicitly adopting the concept of power. We illustrate how different actors, namely, governments, legacy firms, and entrepreneurs, use various forms of power to shape entrepreneurial opportunities. While we argue that the power perspective helps understand how powerful actors might suffocate entrepreneurial opportunities, we also show that entrepreneurs are not powerless actors but can break free from and even modify existing power structures. In short, actors use “power over” entrepreneurial opportunities and have “power to” pursue them as free actors.

## Introduction

Power is everywhere, not because it embraces everything, but because it comes from everywhere (Foucault, 1978, p. 93).

Power may be everywhere, and it is no less ubiquitous in the social sciences and management-related fields, such as organization studies (e.g., Clegg et al., 2006; Fleming & Spicer, 2014; French & Raven, 1959) and strategy (e.g., Hardy & Thomas, 2014). Entire subfields in economics, such as industrial organization, are based on the analysis of market power. The academic discipline of sociology revolves around how power shapes social relations and interactions. Power is one of economic sociology’s four key theoretical lenses (Dobbin 2004). While the other three—institutions, networks, and cognition—have been widely applied in the entrepreneurship literature, power remains underexplored. Moreover, the field of strategy is predicated on how firms can attain and sustain sufficient power to achieve and preserve a strong performance, as are marketing, organizational behavior, and finance. It is hard to find an academic discipline in the social sciences and management that is not preoccupied with the role of power. But not entrepreneurship.

A strange anomaly is that while the concept of power is commonly used across the social sciences and management-related fields, in entrepreneurship, scholars rarely apply a power lens. This striking anomaly may be rooted in the evolution and development of the field itself. As long as the entrepreneurial decision was shaped largely by characteristics and traits of the entrepreneur, power had little to do with what was largely an introspective perspective. However, the era of thinking about the entrepreneur as driven by internal, introspective characteristics, and traits is long gone. Moreover, those studies that explicitly engage with the concept of power tend to focus on how it shapes the social (e.g., Dey & Steyaert, 2016; Lähdesmäki et al., 2019) and economic context of entrepreneurs (Arshed et al., 2014; Welter, 2011; Welter & Baker, 2021).^1^ Furthermore, there is an assertion power may be used against entrepreneurs by actors in an authoritative or privileged position (Baker & Welter, 2020), rather than how power can be used as a resource that actors can activate to shape entrepreneurial opportunities. Power has a transformative capacity (Campbell, 2009), and various forms of power can be utilized by a range of actors, including entrepreneurs.

Power matters for the study of entrepreneurship. For example, the notion that power influences market outcomes dates back at least to Chamberlin (1933) and Robinson (1933) but was more recently adapted by Porter (1979), who argued that powerful firms erect barriers to entry for potential competitors to sustain their market position. While Porter transformed the field of strategy, demonstrating how the distribution of power in a market affects established markets, we know very little about how barriers to entry resulting from the existence of legacy firms affect entrepreneurial opportunities. Christensen (1997) pointed out that incumbent firms may have access to assets and resources that entrepreneurs would struggle to replicate but are also “held captive by their customers” (p. 18), preventing them from finding new applications or markets for their innovations. In contrast, entrepreneurial firms can challenge incumbents with disruptive technologies, shifting power structures in a market. Hence, there are power struggles as legacy firms activate their power to hinder entrepreneurs who would pursue opportunities that challenge existing business models.

This paper provides a pioneering framework of how different forms of power influence entrepreneurial opportunities. We draw on Wrong’s (2017) integration of different forms of power, which allows various power relations to be accounted for. We draw from the existing entrepreneurship literature to show how key actors, including states, legacy firms, and entrepreneurs, activate different forms of power to influence entrepreneurial opportunities. Our coverage ranges widely across countries and social settings. Based on these examples, we discuss how a stronger use of power concepts can contribute to a better understanding of why entrepreneurial opportunities may fail or succeed over time, who can pursue entrepreneurial opportunities, and how such opportunities can be pursued.

An important insight from the literature of sociology is that power differences enable some actors to dominate others (Weber, 1978), so the powerful dictate, suppress, appropriate, allocate, shape, or reshape the conditions for opportunities emerging and being pursued, thereby often restricting entrepreneurial freedoms. Power differences change the value of, and access to, resources (Wijen & Ansari, 2007) and overtly or covertly restrict the distribution of opportunities. Certain actors can hold power over other actors depending on their position in society (Campbell, 2009). In our conceptualization, we are further building on Foucault (1982), arguing that while entrepreneurs are constrained by the power of the social structure, they also have the power to make their own choices (Campbell, 2009). We thus highlight power “comes from everywhere” (Foucault, 1978, p. 96), in different shapes and forms, and can have a transformative capacity. This conceptualization better recognizes that actors can also use power as a resource to enable entrepreneurial opportunities. Thus, we differentiate between two overarching types of power; namely, the power over perspective, which focuses on how actors use forms of power to suppress or restrict entrepreneurial opportunities, and the power to the perspective, which shows how actors can use these different forms of power to realize entrepreneurial opportunities.

Our thesis is that thinking and analysis in the scholarly field of entrepreneurship would greatly benefit from following the social sciences and other management-related fields in considering the role and impact of power, because power dynamics affect the expected outcomes and returns accruing from opportunities and therefore shape the evaluation of opportunities. Section two of this paper draws on the social sciences to expound on the manifold nature of power, investigating three key forms (coercion, persuasion, and authority) relevant to entrepreneurship, two overarching types (power over and power to), and three actor groups (states, legacy firms, and entrepreneurs). In our conceptualization, the state encompasses public organizations (and actors) that have the authority to make binding rules and regulations within their territory (Ingram & Simons, 2000), and influence the allocation of resources in markets (Polanyi, 1957). Legacy firms are organizations that have made substantial investments to create and maintain power. From a resource-based view, their specific investment can be regarded as sunk cost, and because they are difficult to imitate, they can yield a competitive advantage (Peteraf, 1993), but may also create a barrier for further innovation (Christensen, 1997). Entrepreneurs are actors that “endow resources with a new capacity to create wealth” (Drucker, 2014, p. 30).

Sections three and four illustrate each group-type category with poignant examples of how these groups use power to influence entrepreneurial opportunities. While existing studies have seldom explicitly used a power lens, power relationships and dynamics are typically alive and well behind the scene in that they often implicitly underpin streams of entrepreneurship research. Section five provides a discussion and conclusion about how incorporating power into the analysis of entrepreneurship might lead to key insights that otherwise would remain under the grasp of entrepreneurship scholars.

## Forms and types of power influencing entrepreneurial opportunities

### Three forms of power: coercion, persuasion, and authority

The concept of power is not widely used in the scholarly literature on entrepreneurship. However, the opposite holds for most social science disciplines and management-related fields. We adapt from Wrong’s (2017) work in sociology three distinct non-exhaustive forms of power that we see as being important for understanding entrepreneurial opportunities, in that actors can influence others: force/coercion, persuasion, and authority.

#### Force/coercive power

Force/coercive power is defined as the direct use of power for affecting freedom*.* Coercive power imposes on the will of others, typically via legal or other rules, dominance, or sheer force, and affects entrepreneurial opportunities accordingly. Coercion often rests on rewards or punishments (Molm, 1997), whether actual, promised, or threatened. It curtails the freedom of others, including through physical confinement, assault (Goldhamer & Shils, 1939), or psychically affecting another person’s emotions or ideas (Wrong, 2017). For instance, female entrepreneurs may be constrained in pursuing opportunities if facing violence from spouses (Asencios-Gonzalez et al., 2018). Similarly, state laws and policies regulate or restrict firms’ behavior by coercion (Clemens & Douglas, 2006).

Coercive power stems from dependency, as the possession of valuable and scarce resources and knowledge allows actors, including organizations, to coerce the behavior of other actors. By withholding essential resources and knowledge (Fleming & Spicer, 2014) or complementary assets, established rivals can severely restrict or bar even technologically strong entrepreneurs from pursuing opportunities that would disrupt their markets (Braunerhjelm & Svensson, 2010). In addition, states can coerce entrepreneurs through targeted innovation policies and funding to incentivize desired entrepreneurial activity (Carayannis et al., 2017) or offer highly skilled immigrant entrepreneurs special visas (Kerr & Kerr, 2020).

#### Persuasive power

Persuasive power relates to the “tested acceptance of another’s judgment” (Easton 1958; as cited in Wrong, 2017, p. 35). It, therefore, changes the perceptions of others by choice through communication (Wrong, 2017). From the perspective of Foucault (1979), when actors suppress, shape, or produce knowledge through discourse, they exercise power (McHoul & Grace, 2015). Persuasion guides or disturbs the free judgment about opportunities. For entrepreneurs, in particular, effective judgment is seen as critical in recognizing and acting on opportunities (Holt & Macpherson, 2010). Manipulation is essentially persuasion hiding its true intentions (Wrong, 2017). Since discerning manipulation is hard (van Dijk, 2006), we do not dwell on the distinction. Actors vary in their persuasive abilities and access to communication channels such as the mass media. Entrepreneurs may need to resist persistent persuasion (Artinger et al., 2015) to maintain their judgment of opportunities (Kirzner, 1973), including persuasion campaigns by larger businesses (Gans & Stern, 2003) and governmental states (Fiedler et al., 2021), while also deploying their own persuasive powers: “Being able to persuade stakeholders to invest into their idea and to buy from them is the sine qua non for starting a business” (Artinger et al., 2015, p. 739). Thus, persuasive skills, both offensively as well as defensively, matter for entrepreneurs (Baron & Markman, 2000; Baron & Tang, 2009).

#### Authoritative power

Authority flows from the perceived personal attributes of the power holder (French & Raven, 1959). It rests on the untested acceptance of a command or judgment by virtue of formal roles, achievements, resource endowments, specialized knowledge, or even charisma. As subjects often defer to holders’ reputation—for example, for expertise: being “an authority”—authority generally implies consensus (Wrong, 2017). Succinctly put, while both persuasion and authority affect judgment about opportunities, the former implies that the judgment is tested while the latter does not.

In judging opportunities, entrepreneurs can be subjected to different overlapping sub-forms of authority. Examples would include individual actors that have power either through their formal role endowing them with coercive forms of power, or they have informal power, because of their personal characteristics and qualities, such as expert knowledge or charisma (adapting Raven, 2001). To illustrate, venture capitalists have authority over entrepreneurs because entrepreneurs must surmount information asymmetries and signal the venture’s potential (Busenitz et al., 2005)—but they are not essential for the pursuit of the opportunity, as there is no direct dependency between the two groups of actors, neither can they guarantee success. Entrepreneurs, too, can activate authority; for instance, legitimate authority over employees in how they reward human capital when pursuing the opportunity (van Praag & Versloot, 2007), or a reputation for competent authority when investors and other stakeholders perceive them as experts through their entrepreneurial and industry experience (Dimov, 2010).

### Two overarching types: power over and power to

Power influences the behavior of free actors. It constitutes a possibility of action and therefore often influences indirectly and modifies the action of others by its capacity to react to their action. It is often a response (Foucault, 1982). The entrepreneurship literature recognizes the centrality of entrepreneurial actions for opportunity development. Entrepreneurial action is influenced by cognitive and emotional factors, such as feelings and knowledge, and by the freedom to act on perceived opportunities (Kuratko et al., 2021). It can be assumed that power structures will affect opportunities. Juxtaposing entrepreneurship and power suggests that an entrepreneurial action will lead to confrontation, struggles, and transgressions.

We distinguish between two non-mutually exclusive types: power over and power to (Göhler, 2009; Pitkin, 1972). We bring together forms and types of power for three key actor groups: the state, legacy firms, and entrepreneurs. Table 1 outlines our differentiation between actors exerting power over opportunities and exercising power to pursue opportunities. Power over refers to the restricting or influencing of behavior and judgment of others about the opportunity they pursue (how people think about opportunities and which can be pursued without reprimand), while power to refers to use of power in an actors’ own pursuit. Power to also highlights power’s transformative capacity (Campbell, 2009), when individuals have an ability or capacity to somewhat ignore power over (Philp, 1983).

**Table 1:** Examples of types and forms of power for key groups of actors

Group of actors	Form of power	Example*
Type: Power over entrepreneurial opportunities (how people think about opportunities; and which can be pursued without reprimand)		
State	Coercion	Regulating who can pursue entrepreneurial opportunities in given contexts, such as the crackdown on education-technology firms in China
	Persuasion	Dominating narratives about entrepreneurial opportunities, such as encouraging focus on growth
	Authority	Corrupt officials illegally rely on entrepreneurs’ perceptions of the powers of their office to extort bribes, for example, permits, imports licenses, or government contracts
Legacy firm	Coercion	Forcing network partners to adhere to certain otherwise-optional industry standards, such as certifications—for example, to comply with certain norms
	Persuasion	Enticing entrepreneurs to sign exclusive licensing agreements to maintain market dominance
	Authority	Developing a daunting reputation for how the firm will mobilize resources to react to entrepreneurial challengers—for example, by acquiring or copying them
Entrepreneurs	Coercion	Coercing R&D-intensive firms to pay an unanticipated license for a patent held by a coercing entrepreneurial firm without the intention to develop their own product or service
	Persuasion	Creating stories to persuade investors to re-allocate resources
	Authority	Creating a personal reputation within society related to innovation, such as Elon Musk being “The Dogefather” of the cryptocurrency Dogecoin
Type: Power to pursue entrepreneurial opportunities (use of power in actors’ own pursuit)		
State	Coercion	Channeling targeted incentives, such as R&D grants
	Persuasion	Encouraging actors to join government-led initiatives that respect their pursuit of entrepreneurial opportunity, such as becoming part of an ecosystem
	Authority	Agencies and agents gaining expert reputation regarding entrepreneurial opportunities, such as government-led consultancy services
Legacy firm	Coercion	Dominating entrepreneurial opportunities in certain markets, such as an establishing platform like Amazon
	Persuasion	Lobbying government affiliates to support services, such as Rural Taobao
	Authority	Developing an entrepreneurial image, such as Google to be an innovative firm
Entrepreneurs	Coercion	Asking network partners to share risk and resources
	Persuasion	Creating stories to promote innovation, such as Thomas Edison did
	Authority	Creating an aura of expertise, vision, and coolness

The aim in the following section is to empirically illustrate how use of power affects entrepreneurship. To demonstrate the potential of power analysis, we illustrate each type-actor-form combination. We illustrate power over before power to, because the latter can countervail the former, which often hinders rather than promotes opportunity pursuit. Mindful that power “comes from everywhere” and is not restricted to those who can impose the law on others but can be productive and bring things into being (Foucault, 1978, p. 93). We do not claim that our categories or illustrations are mutually exclusive, exhaustive, or cover all interrelationships, but they render transparent the as-yet opaque importance and neglected potential of the concept of power for entrepreneurship.

## Exerting power over entrepreneurial opportunities

### States

#### Coercive power

Promoting entrepreneurship, power inequalities between large organizations and entrepreneurial firms (Nicholas, 2003) legitimize states’ coercive power to enable entrepreneurs’ pursuit of opportunities. While policy instruments including regulation coerce and constrain the behavior and power of market actors, “taxes, incentives, subsidies, and grants” reward desired behavior (Cohen, 2006, p. 4). State intervention often lets entrepreneurs pursue opportunities by shielding them from the power of legacy firms. Thus, the Sherman Act, which was enacted in 1890 as a foundation for US antitrust law, or competition policy, provides the legal basis for constraining powerful organizations asserting or misusing their market power by fixing prices or monopolizing the market (Golodner, 2001). As Senator John Sherman admonished the US Congress, “If we will not endure a king as a political power, we should not endure a king over the production, transportation, and sale of any of the necessaries of life.” Guerrero and Urbano (2019) provide compelling examples and analysis of how international entrepreneurs receive support in the form of credit guarantees and loan subsidies from governmental states to counteract resource constraints.

Conversely, coercively hindering entrepreneurship (Cohen, 2006), governmental states can regulate and restrict who may pursue what opportunities and under which conditions. For example, governmental states have restricted women’s property rights and movements “outside of the house,” effectively reducing entrepreneurial opportunities for women (Estrin & Mickiewicz, 2011).

Furthermore, governmental state actors, such as regulators and even courts, can serve their own interests by protecting what is termed in the political science literature as constituting legacy firms (Gurses & Ozcan, 2015) that may lobby, fund, or belong to them, or simply promote the state’s interest at the expense of entrepreneurs. Extreme governmental state power over opportunities for political ends can stifle entrepreneurial efforts. For example, China’s recent crackdowns on privately owned technology companies illustrate this. Education-technology entrepreneurs were banned overnight from making profits or raising funds on stock markets, justified as trying to reduce student workloads (CNN Business Staff 2021; Mumme, 2021). Similarly, state media labeling online gaming as constituting “spiritual opium” forced gaming companies, such as Tencent, to limit the time children can play online (Sweney & Davidson, 2021). Most striking is the recent fall of Jack Ma, founder of Alibaba, whose products hundreds of millions of Chinese citizens use daily. The Financial Times (McMorrow & Yu, 2021) reported that Ma, with his “cult-like following… became too powerful in a country that only allows a single center of power,” with threats “to crush him” as he started to challenge state power. Reportedly, the Chinese Government has singled out other individuals and influential entrepreneurs, such as film stars and entrepreneurial Zhao Wei, who have not conformed with government narratives and shut their businesses (Seidel, 2021). Such selective state coercion of individual entrepreneurs and sectors, reflecting the exercise of governmental power to the detriment of entrepreneurs, deserves more attention for understanding entrepreneurial opportunities.

#### Persuasive power

The existing literature makes it clear how states can deploy high persuasive power, amplified by media access and control, over entrepreneurial opportunities to achieve their goals while minimizing risks of opposition. Perren and Jennings (2005) illustrate how states shape discourse to steer entrepreneurial judgment by telling them “to provide ‘economic’ returns (Australia), to “grow” (Japan, Korea, [United Kingdom] UK, US), to “[stand] tall in their own right” (Korea) and to provide a “steady basis” of growth so that the national economy can progress (Korea)” (Perren & Jennings, 2005, p. 177). Discourse, though, might mismatch and ill-serve entrepreneurial capabilities and interests and instill a false judgment of opportunity (Fiedler et al., 2021). Audretsch and Fiedler (2022) have shown how the policy accord of entrepreneurial states can impose a knowledge filter within society that can suppress certain entrepreneurial opportunities. Thus, while some existing studies suggest that states use persuasive power to encourage entrepreneurial opportunities to pursue their own goals, more research is needed to understand better how entrepreneurial opportunities are negotiated in discourse between state actors and other stakeholders, including how government opposition and larger firms might counterbalance the dominant narrative.

#### Authoritative power

Actors might lean on state officials with perceived superior access to resources or knowledge in judging opportunities. Take corruption. Officials may require bribes to access critical resources, for example, permits for business operations (Audretsch et al., 2021), import licenses, or government contracts (Smith, 2016) (Table 1). This restricts opportunities. Corruption significantly burdens entrepreneurs, raising the cost of doing business and wasting time (Agboli & Ukaegbu, 2006). As authority relates to the personal attributes of the power holder or the office they hold, it is intersubjective, and variations occur within a given context.

How the exercise of authoritative state power affects entrepreneurial opportunities over time is not well understood. It has been long assumed the formation of an entrepreneurial class would be a driving force for democratization, reducing the authoritative power of state actors, but evidence from late-developing economies indicates this may not be the case (Audretsch & Fiedler, 2021). Tsai (2005) calls for a more fine-grained investigation regarding entrepreneurial backgrounds and their social context to understand how they are affected by authoritative states, as some entrepreneurs might benefit from relationships with powerful political actors and thus have no incentive to drive democracy. Furthermore, Gan and Xu (2019) show that in China, local officials’ corruption misaligns with central government goals, as firms headquartered in regions with stronger anti-corruption efforts spend more on R&D. To counterbalance, the central government’s anti-corruption campaign (Gan & Xu, 2019) partly aims to stimulate entrepreneurial opportunities.

### Legacy firms

#### Coercive power

Legacy firms can wield coercive power over opportunities to appropriate them or shut them down. Entrepreneurial firms often depend on larger organizations’ support and goodwill due to power inequalities—for example, for financing or access to key resources (Hancké, 1998)—including depending on multinational corporations (MNCs) and state organizations that have power over them due to structural disparities like asymmetric market power. Larger firms that control critical resources can also coerce behavior and procedures or standards of production (Guler et al., 2002) in ways that confine opportunity. Gans and Stern (2003) show that technology entrepreneurs need to consider power relationships in their industry. Only if entrepreneurs could overturn the assets of established players and independently lead technology change could they compete against incumbents; if established firms kept control of complementary assets, entrepreneurs had to seek cooperation, which then protected the legacy firm’s market power. Theories on social exchange, such as Emerson’s (1962) power dependency, can provide a suitable lens through which to investigate how power inequalities influence relationships between large firms and entrepreneurs when one of the parties controls a resource valuable to the other (Blau, 1964).

Legacy firms can coerce entrepreneurs even without controlling key assets. Venture capitalists speak of a “kill zone,” when powerful tech companies crush or buy innovative start-ups that challenge their business models. Notably, Facebook Inc. acquired smaller entrepreneurial ventures, including Instagram and WhatsApp Inc., and imitated features from Snapchat (McLeod, 2020). The entrepreneurship literature has paid scant attention to such complex firm-level power relations and their impact on entrepreneurial opportunities. One useful analysis (Fligstein, 2003) points out that legacy firms embedded in Silicon Valley, including Microsoft, Intel, and Cisco, have all been defendants in predatory practice antitrust cases.

Reasons why legacy firms restrict the freedom of entrepreneurs to discover and create opportunities in their industry often stem from their cost structures and existing customers. Legacy firms have invested in structures, platforms, knowledge, and technology, or sometimes deliberately overinvested to deter entry (Fudenberg & Tirole, 1984). The resulting fixed and sunk costs mean innovation or disruptive technologies, or businesses models, could crowd them out, besides displeasing customers wedded to existing products (Christensen, 1997). Legacy costs make entrepreneurial ventures look threatening. Instead of embracing purposeful change by letting them compete, legacy firms tend to protect the status quo by leveraging their power to distort access for entrepreneurs to pursue opportunities.

#### Persuasive power

Legacy firms might exert persuasive power over opportunities. In the network relationships described, the boundaries between coercive and persuasive power can blur. Powerful legacy firms may use persuasion—including, when intentions are hidden, manipulation—by incentivizing entrepreneurs to join their network as members, not competitors. For example, strategically licensing legacy technology to entrepreneurs early on preempts independent R&D that could disrupt industries and erode the legacy firm’s dominance (Gallini, 1984). Legacy firms have also selectively licensed their patents to multiple small entrepreneurial firms to engineer an industry structure of powerless competitors, which may constitute an oxymoron (Rockett, 1990).

Legacy firms may use persuasion, or manipulation, when sharing information with entrepreneurs to influence judgment about opportunities. For example, they may lure entrepreneurs in possession of valuable technology into an exclusive licensing agreement, tethering the entrepreneur (Somaya et al., 2010). Similarly, in technology partnerships between smaller and larger firms, hidden agendas abound (Doz, 1987). Exchanging information and ideas, alongside formal contracting, can build trust in the partners’ intention to establish a mutually beneficial technology collaboration (Walter et al., 2015). Yet communication might belie actual behavior and conceal true intent. From a power perspective, licensing strategies that are aimed at weakening entrepreneurial competition constitute manipulation: the legacy firms hide their goal of sustaining dominance, so that the licensing provides a type of Trojan Horse. Also, when R&D alliances and partnerships fail, the entrepreneur risks losing their technology to their larger partner, and ultimately, their competitive advantage (Ahern, 1993), suggesting that power relationships might change over time as actual outcomes may not be aligned with expectations. Nonetheless, researchers rarely apply a power lens to investigate how legacy firms might manipulate entrepreneurial opportunities.

#### Authoritative power

Building on actual displays of coercive power such as in the kill zone, legacy firms have developed a daunting reputation as to how they will mobilize resources, and that reputation enables some authoritative power over opportunities (Table 1). Legacy firms that react to challengers by collaborating, acquiring, or copying entrepreneurial firms inhibit entrepreneurial strategies. This affects entrepreneurial judgment about the opportunity. For example, the cost of establishing and defending patents may outweigh the benefits for small firms (Athreye 2021). Partly that is because the daunted small firms (mis)perceive that legacy firms have the authority to “invent around” a patent, registered design, or technical specification (Hughes & Mina, 2010). There is also a perception that legacy firms will acquire patents from entrepreneurial ventures to maintain market power and keep competition away (Salant, 1984). All these power projections might deter smaller firms from patent licensing.

### Entrepreneurs

#### Coercive power

At first glance, entrepreneurs hold scant coercive power. Thus, most see little room to exercise coercive power over other actors’ opportunities and change the “rules of the game” in their own favor (Santos & Eisenhardt, 2009). Entrepreneurial small business owners typically cleave to local norms because they depend on local community support (Lähdesmäki et al., 2019). Yet, entrepreneurs might coerce some stakeholders in developing opportunities, thus exerting that power over other entrepreneurs. An extreme case is what Volkov (2016) calls “violent entrepreneurs”: certain social groups in Russia secured ongoing financial and other market resources by organized violence. Specifically, they licensed out memberships. Members could use the group name as a “trademark,” giving them protection and enforcement services. These violent entrepreneurs created a market for opportunities when the Russian state failed to exert coercive power to protect the civil rights and basic safety of its citizens. Another example is patent sharks (Table 1). These entrepreneurs exert power over R&D-intensive firms by betting on certain technology, acquiring patents that they never intend to use for manufacturing but instead hold to obtain licensing income (Reitzig et al., 2007).

#### Persuasive power

Entrepreneurs may exert persuasive power over access to opportunities by influencing others. Effective communicators can foster big organizations’ loyalty and trust (Woldesenbet et al., 2011), mitigating power inequalities. Highly persuasive entrepreneurs who can better mobilize a network, and influence the judgment of others even with rather weak ties, need not utilize other resources to launch their venture. Persuasive communication paves access to critical resources—for example, raising potential investors’ interest (Clark, 2008). Powers of persuasion will vary among individual entrepreneurs. Comparing persuasion techniques during price negotiations suggests entrepreneurs might be less skillful than non-entrepreneurs and less likely to close deals; they focused on particularly profitable opportunities and negotiated hard, relying too much on contra-arguments (Artinger et al., 2015).

#### Authoritative power

From a social capital perspective, while entrepreneurs at least usually start with little economic and political power, they often enjoy strong social relationships with their network partners, conferring authority within their network (Fuller & Tian, 2006). Some have successfully used their authority, such as the personal authority of charisma and expertise, or competent authority, and persuasion, to exert power over other actors. Take Elon Musk, named “The Dogefather” (after Dogecoin), whose social media tweets have been enough to lift cryptocurrency values due to his visionary status (Table 1) (Turner-Cohen, 2021). Audia and Rider (2005) argue that successful entrepreneurs such as Steve Jobs gain power over opportunities from connecting emotionally to the public. Furthermore, the very community support that made Lähdesmäki et al.’s (2019) norm-obeying small business entrepreneurs refrain from coercion for fear of alienating others can constitute trusted authority within their network, governing power relationships with strong emotional ties.

## Exercising power to pursue an entrepreneurial opportunity

### States

#### Coercive power

States can exercise coercive power to recognize and create opportunities that might then be pursued by entrepreneurs. While the industrial policy literature has long explored the state’s role in shaping the institutions in which private actors enact entrepreneurial opportunities, more recently, Mazzucato (2011) has argued an “entrepreneurial state” can also actively create, direct, and exploit entrepreneurial opportunities. Both approaches concern how states may coercively shape opportunities, but Mazzucato’s contemplates a more active role, moving beyond “defining the rules” to actually “playing the game.”

States can use coercive power to implement policies that increase or channel the knowledge available to entrepreneurs, such as policies related to labor mobility, R&D investment, and patents. These policies may stimulate firm formation (Choi & Phan, 2006). A prominent example is policy designs to encourage knowledge spillovers, such as incentivizing inward foreign direct investment (Acs et al., 2012), or attracting migrant or returnee entrepreneurs (Liu et al., 2010; Si & Bruton, 1999). Economic development strategies of emerging markets like China have attracted foreign firms to enable just such knowledge spillovers and technology upgrading, supporting domestic firms (Li et al., 2013). This involves coercively limiting how foreign firms can operate in their constituencies, including limits on ownership and governance structure (Osland & Cavusgil, 1996). Knowledge spillovers can be leveraged by entrepreneurs embedded in the same context to create opportunities (Agarwal et al., 2007).

States may also use coercive power to focus opportunity discovery and creation in key areas. For instance, the entrepreneurial state may limit certain activities to channel entrepreneurial resources along with ancillary activities into sectors it deems more desirable and productive. For example, Singapore set a minimum age for its citizens to engage in the gig economy as ride-hailing drivers, to direct younger people to develop skills in more productive areas (Ruehl, 2021). Here, coercive power is commensurate with power to.

#### Persuasive power

States can use persuasive power to create, direct, and exploit opportunities and foster entrepreneurship. Policy design to foster entrepreneurial ventures in their innovation efforts is often accompanied by supporting arguments, as Hughes (2009) explains. For instance, UK policy innovations to aid small to medium-sized enterprises (SMEs) are justified by widely shared assumptions about market failure, such as SMEs’ lack of access to skilled employees or risk capital. These arguments then serve to carry broader innovation-support programs to spur innovation in entrepreneurial ventures, such as R&D grants (the grants themselves are an example of coercion in Table 1), small firm R&D tax credits, and public-sector R&D procurement (Hughes, 2009). The policies that allocate resources are coercive, and the arguments are persuasive.

States have also used narratives to inspire citizens to become entrepreneurs. Israel’s narratives have shifted from ancestral pioneers who settled on the land to today’s pioneers: high-tech start-up entrepreneurs transforming Israel into a “start-up nation” (Senor and Singer 2009, as cited in Fraiberg, 2017). Jessop (1998) pointed out that states cast narratives related to their self-image linked to entrepreneurial opportunities, including the “competition state,” the “entrepreneurial city,” and the “learning region,” to draw entrepreneurs and capital and promote entrepreneurial growth. Hence, the state uses persuasive power to improve citizens’ perception of entrepreneurial opportunities deemed desirable and to engage them in innovation.

#### Authoritative power

States or their agents may gain authoritative power to influence entrepreneurship. Public servants, for instance, increasingly mold and flag opportunities, for example, through government-led consultancy services (Table 1). Agencies in many countries supply knowledge and information about business-growth opportunities, such as export support (Sousa & Bradley, 2009) and innovation-support agencies. Interestingly, managers of an innovation program in a peripheral region of the UK lost authority by failing to recognize the full range of innovation activities of some participating SMEs and the benefits of forms of innovation beyond R&D (Galbraith et al., 2017). So, to be perceived as a trusted source of advice for the discovery/creation of entrepreneurial opportunities, governments, and their officials face the challenge of establishing and maintaining authority.

More successful has been South Korea’s information and communications technology (ICT) transformation. Officials trained—often at top US universities—in related fields, such as economics, were pivotal in moving the country towards pursuing more opportunities in science and technology (Larson & Park, 2014).

### Legacy firms

#### Coercive power

Legacy firms may exercise coercive power to shape entrepreneurship. Some business models, for instance, let legacy firms coercively control opportunities and the ecosystems that home them. For example, take online digital platform providers, such as Amazon, Google, and Facebook (Cutolo & Kenney, 2020). While their platforms provide channels and related services to empower individuals to build a business, the overall power to define the opportunity remains firmly with the provider. Likewise, albeit while retaining ultimate control, the online marketplace, and logistics network Amazon (Table 1) has revolutionized e-commerce. Isckia and Lescop (2009) point toward Amazon’s self-image as an incubator for entrepreneurial firms, facilitating opportunities and empowering American SMEs to grow through ICT-based open innovation. Similarly, Alibaba has supported entrepreneurs’ pursuit of opportunities, for instance, by advancing micro business loans to three million entrepreneurial SMEs (in 2018) through their service Ant and enabling poor rural residents to engage with e-commerce through their Taobao Village strategy in 2014 (Kwak et al., 2019; PYMNTS, 2021; Zeng, 2018).

However, the case of these platform enablers also shows how power disparities blur the boundary between the power to enable new opportunities and power over opportunities. Cutolo and Kenney (2020) term entrepreneurs engaged on digital platforms as platform-dependent, arguing that power imbalances are intrinsic to the platforms’ design by larger players. Similarly, Krugman (2014) argues that “*Amazon … has too much power, and it uses that power in ways that hurt America.*” He claims Amazon is acting as a monopsonist, or dominant buyer, pressuring sellers to cut margins and disrupting the business of non-co-operators—for example, by delaying their delivery or redirecting the customers’ attention to competing sellers. Khan (2017) maintains that Amazon remains dominant because current antitrust law focuses on customer welfare related to how the lack of competition may influence prices and outputs.

#### Persuasive power

Legacy firms may use persuasive power when lobbying other actors, notably the state, to set rules that favor the opportunities they themselves judge desirable. Theories of regulatory capture describe how and why governments become subservient to the power of private firms and interests. Investments in policy influence can build barriers for entrepreneurs to compete, protecting rents of investments of legacy firms (Dal Bó, 2006; Laffont & Tirole, 1991). Legacy firms may also use persuasive strategies to gain support from the state to pursue opportunities. Zhang (2020) reveals how Alibaba (now a legacy firm) collaborated with both central and rural governments to establish the mentioned Taobao Villages venture, bringing e-commerce opportunities for rural entrepreneurs. Lobbying agencies, research institutions, universities, and other actors, all government-affiliated, produced proposals sent from supporters to parliamentary representatives. This raised awareness of rural e-commerce and led “*the state to issue a series of documents to encourage rural e-commerce*” (Zhang, 2020, p. 125).

#### Authoritative power

Legacy firms also rely on authority to create entrepreneurial opportunities. It has been said legacy firms often lose their ability for visionary innovation. But some large tech firms recognize they must keep innovating and adjusting to market dynamics by encouraging entrepreneurship through internal processes (Christensen, 1997), such as corporate entrepreneurship (Guth & Ginsberg, 1990). Finkle (2012) found corporate culture was the key to Google staying innovative and stimulating entrepreneurship. It cultivated the image that its mission was “to improve the world” through innovation and entrepreneurship, projecting authority” (Finkle, 2012, p. 879).

### Entrepreneurs

#### Coercive power

As also noted under power over entrepreneurs can often exercise only limited coercive power to pursue an opportunity—for instance, if they hold a sufficiently valuable innovation to disrupt an industry, forcing a new business model industry because of the absence of legacy cost (Table 1). However, Sarasvathy’s (2001, 2009) work on effectuation illustrates different approaches entrepreneurs use to gain control over opportunity development. Unlike the causal approach that sets a goal and works backward to what is necessary to achieve it, effectuation asks what can be made using existing means. Effectual entrepreneurs then use a logic of non-predictive control, focusing on their identity, knowledge, and networks. Power flows from several mechanisms as they take only calculated risks, based on the affordable loss principle, and only take on network partners that commit to co-develop opportunities and share risks (Sarasvathy et al., 2014). Applying the effectual principle implicitly considers power structures, since control and power often go together.

Effectual entrepreneurs are less exposed to power disparities or external authority because they focus on opportunities they can control rather than predicting the future (Sarasvathy, 2009). Insisting that network partners share risk also reduces the potential for partners to activate power over them.

#### Persuasive power

Entrepreneurs can certainly draw on persuasive power to pursue opportunities and countervail power exerted over them. Ways to persuade stakeholders to commit resources to the entrepreneurial venture and support their opportunity include storytelling (Table 1) (Lurtz & Kreutzer, 2014; Sole & Wilson, 2002) and using metaphors to win support for novel venture ideas (Cornelissen et al., 2012). Geissinger et al. (2019) illustrate persuasion strategies a Swedish entrepreneurial venture, Comvik, used to navigate competitive pressures from a legacy firm, Televerket, a publicly owned monopoly that had granted Comvik a concession to operate in a small niche of the mobile telephony market. For example, when new technology emerged, Televerket tried to exert coercive power over the entrepreneur by banning Comvik from adopting these new technologies. Comvik mobilized the media and public authorities to call out this anticompetitive behavior, such as by publishing an article that accused Televerket of “misus[ing] their power as regulator” (Geissinger et al. 209, p. 880) and deployed the David and Goliath metaphor. These strategies influenced the authorities to rule in the entrepreneur’s favor, preventing regulatory capture.

American inventor and tycoon Thomas Edison provides another example of how entrepreneurs have used persuasion. To promote a direct current (DC) electricity system, Edison graphically demonstrated the lethality of Westinghouse’s rival alternating current (AC) system by staging electrocutions of animals in a public campaign (Cole & Chandler, 2019).

#### Authoritative power

Entrepreneurs may activate authoritative power to pursue opportunities by leveraging their personal characteristics during opportunity development. For example, personal authority can result from individual characteristics such as entrepreneurial identity (Singh et al., 1986), a formal degree such as a PhD (Hsu, 2007), social values (Newth & Woods, 2014), or passion for growth. All can complement or even compensate for other forms of power (Lewis & Cardon, 2020). Entrepreneurs enjoy a positive, even heroic image (Johnsen & Sørensen, 2017), sometimes romanticized as sole agents of change (Drakopoulou Dodd & Anderson, 2007). Some, like singer Jennifer Lopez, can leverage celebrity capital as a strategic resource to pursue entrepreneurial opportunities, such as Lopez’s beauty and clothing lines. Entrepreneurs who became celebrities because of their success at establishing ventures, such as Richard Branson’s association with Virgin Airlines, may signal entrepreneurial skills valued by others that yield unique opportunities for their new ideas (Hunter et al., 2009).

## Discussion

Power matters for entrepreneurial opportunities. Unlike academic fields throughout management and the social sciences, entrepreneurship literature rarely makes power a focal lens. Studies that have investigated power more explicitly (e.g., Baker & Welter, 2020; Ramoglou et al., 2021) tend not to embrace the multifaceted nature of the concept. Building on Foucault (1978), who argued that power can come from everywhere, we have re-examined the extant entrepreneurship literature to detect and illustrate how different forms of power shape opportunities and the judgment about them. We used a conceptual framework that focuses on (a) three forms of power (coercive, persuasive, and authoritative); (b) three groups of actors (states, legacy firms, and entrepreneurs); (c) two overarching types of power (power over and power to) to restrict, enable, or guide entrepreneurial opportunities. In doing so, we offer an introductory conceptualization of how the concept of power matters for entrepreneurial opportunities and we aim to stimulate debate.

The power over perspective highlights power flowing from control over key resources. Such a view can help us grasp how opportunities are judged and shaped over time, and particularly what limits may exist within a society or market on entrepreneurs’ ability to challenge and disrupt existing power structures. The power over perspective tends to reveal more restrictions than promotions of opportunities and has limits in explaining how entrepreneurial firms commercialize disruptive technologies that may displace more powerful incumbents (Carayannopoulos, 2009). In fact, power often serves to maintain established structures, forgoing new entrepreneurial opportunities, which can come at a substantial welfare loss. Thus, a power-over lens can complement institutional perspectives on entrepreneurial failure (Lee et al., 2021) and shed new insights as to why entrepreneurs may not meet their growth potential or even fail, despite superior technology.

The power to perspective better explains why and how entrepreneurial firms have defied existing power structures. While entrepreneurs are often seen as powerless actors, they still have the power to mobilize and influence other stakeholders to realize their opportunities (Ramoglou et al., 2021). By activating power resources, in particular noncoercive forms such as authority that often grant them soft power (Nye, 2011; Santos & Eisenhardt, 2009), entrepreneurs may evade, counteract, or challenge existing power structures and be a productive source of welfare gain. Thus, the power to perspective can explain how actors free themselves from existing power structures and take control of opportunities—a hallmark of entrepreneurship.

Our analysis also suggests ways power over and power to interact and might even be mutually constituting. For example, if legacy firms overuse their power over others, entrepreneurs may have the power to counter and create or defend a judgment about an opportunity by mobilizing fellow actors, often by persuasion, to support redistributing power in their favor. Both states and legacy firms can use coercive power to and power over to influence entrepreneurial opportunities but must maintain other sources of power to avoid resistance, confrontation, and similar undermining reactions. As Foucault noted (1982), power is often a response. If powerful actors lose authoritative and persuasive bases for their actions and rely solely on coercive forces, other actors may respond by counteracting them.

We see three key features of power analysis that can make connections between power and the development of entrepreneurial opportunities more explicit. Namely, power (a) is interaction focused, (b) reveals itself after key events during episodes (Lawrence, 2008), and (c) has boundaries that may not correspond to geographic or institutional boundaries. Based on these three features, we now outline the promise of power for studying entrepreneurial opportunities.

First, power is interaction focused (Fleming & Spicer, 2014). It highlights the dynamics between the powerholders and those that are subject to it. Investigating such interactions as the basis for analysis can reveal key actors that shape opportunities. Building on Kirzner (1973), Shane and Venkataraman (2000) argued opportunities exist because different actors vary in their perception about the relative value of resources, and during the entrepreneurial discovery process, resources are put to a better use for value creation. A power lens can complement this perspective because power directly affects both the access to key resources and judgment about opportunities.

Our analysis suggests that entrepreneurial opportunities might be (overtly or covertly) suffocated, or enabled, by powerful actors, including those who control key resources. Thus, power differences might also explain why certain actors, such as minority entrepreneurs, are not pursuing certain entrepreneurial opportunities. Since Schumpeter first recognized entrepreneurs as central actors for economic growth (Baumol, 1968), entrepreneurship scholars have highlighted differences between characteristics not only of entrepreneurs and non-entrepreneurs but also of entrepreneurial subgroups, such as migrant entrepreneurs (Kloosterman, 2010), female entrepreneurs (Brush & Cooper, 2012), technology entrepreneurs (Rojas & Huergo, 2016), and social entrepreneurs (Newth & Woods, 2014). These subgroups are affected by power. For instance, state-imposed visa conditions that preclude migrants from running their own business (Cohen et al., 2011), coercively limiting who has the right to be an entrepreneur. Legacy firms, on their part, may disguise how they activate their power over entrepreneurial opportunities by embedding emerging technology ventures into their existing network structure through resource access to avoid regulation.

A focus on interactions can also reveal how power structures shift. Entrepreneurship can be a mechanism for some actors, including female entrepreneurs, to break free from existing power structures, thereby triggering social change and modifying power structures (Baikovich et al., 2021). Furthermore, entrepreneurs are resourceful at circumventing burdensome regulation (Shleifer & Vishny, 1993) imposed by powerful actors. They may challenge or break rules. Indeed, entrepreneurs might be better at rule breaking (Kuratko & Goldsby, 2004; Williams & Gurtoo, 2011) than powerful actors, having neither legacy cost nor customers to alienate. Thus, while entrepreneurs are subject to power, depending on their background as well as the context in which they operate, they might carefully navigate power structures and defy powerful actors in the pursuit of opportunities. Here, power also offers a framework to understand better the temporal boundaries of the relevant context for entrepreneurial opportunities (Welter, 2011), because a power perspective can support our understanding as to what mechanisms can be used to shape context relevant to entrepreneurial opportunities.

A power lens might also provide new insights into studies on entrepreneurial networks. While previous research has recognized the development of entrepreneurial opportunities requires support and engagement from various stakeholders (Bosse et al., 2022), highlighting the role of supportive entrepreneurial network partners, such as family, friends, and business partners (Hoyte et al., 2019), viewing networks as a potential resource to be cultivated may gloss over underlying power dynamics. The study of power considers harmful actors. Power is unevenly distributed, and actors, within and outside a relevant network, can use different forms of power against entrepreneurs. Power relations are subject to change. Shifting coalitions can influence entrepreneurial opportunities. By investigating the interactions within entrepreneurial networks through a power lens, researchers can gain new insights into the mechanisms through which entrepreneurial networks evolve over time and what strategies entrepreneurs can use to navigate existing power structures.

Second, a focus on episodes of action can bring power dynamics to the fore and help us to understand how key events might trigger changes in power structures (Lawrence, 2008). Recently, we have seen how the COVID-19 pandemic (Audretsch & Moog, 2022) and war (Brück et al., 2011), such as the Russia–Ukraine war, has destroyed entrepreneurial opportunities, suggesting that certain episodes of action of powerful actors can be destructive forces to entrepreneurship. Nonetheless, some entrepreneurs might even seize an opportunity from crisis and conflict. For example, Giunipero et al. (2021) find during the COVID-19 pandemic, some small businesses benefited from power shifts in the supply chain, allowing them to pursue new opportunities. Also, drawing on case studies of female entrepreneurs during the Niger Delta Conflict, Anugwom (2011) finds the collapse of the traditional economy resulted into niche opportunities for women. They became a critical actor to provide services for militants in the context of war, not only securing them an income but also allowing them to assemble a network that may remain relevant for opportunities in the future. War may also provide opportunities for actors to engage in social entrepreneurship; for example, by becoming engaged in collective action related to peace-building activities (Brück et al., 2011). Thus, early studies show crisis and war shifts the impact of power structures on entrepreneurial opportunities.

Similarly, events on the individual level, such as meeting with potential investors, or the industry level, such as the entry of new competitors, including legacy firms that move into the entrepreneurial space, may shift power relations between entrepreneurs and other actors. For example, Fath et al.’s (2021) study track three different pathways of opportunity development after competitors emerge. The study shows the emergence of competition was a critical event for entrepreneurs, making them aware of power inequalities within their industry, which then affected their perception of the entrepreneurial opportunity and how they further developed their network. Future studies are needed to explore how critical events and episodes affect opportunities. Grasping the changing power dynamics between actors after episodes of strategic action is important, as it may open niche opportunities and affect power structures relevant to entrepreneurs and result in changes in entrepreneurial strategies.

Third, the power perspective highlights boundaries of influence. Recently, interest has risen in regional studies, including entrepreneurial ecosystems (Theodoraki & Catanzaro, 2021) and cities (Audretsch et al., 2019) that shape entrepreneurship. Existing studies suggest, depending on the wider cultural context, different tolerances for power inequalities within ecosystems or cities exist (Audretsch & Fiedler, 2021), as well as different tolerances exist for power inequalities between actors such as entrepreneurs and states (Tsai, 2005). Comparative research could use a power lens to explore how entrepreneurial opportunities are shaped in different cultures. Furthermore, while power is a concept rooted in a specific context, that context can transcend, and might not correspond to regional boundaries. An illustration is Silicon Valley. While many studies analyze how entrepreneurial opportunities have evolved within its ecosystem, others suggest its influence on entrepreneurial opportunities goes well beyond regional (subnational) or even national boundaries (e.g. Whittaker, 2009). Future studies are needed to investigate the power structures of a specific place that affect beyond that place, including the impact of the internet as a boundary-breaching space.

In concluding our argument as to why power matters for entrepreneurship—the judgment about opportunities and the freedom to pursue them—we would like to return to Foucault, who argued that “*power is exercised only over free subjects, and only insofar as they are free*” (1982, p. 790). There is an increasing interest in the role freedom plays in encouraging and shaping entrepreneurship (Audretsch & Fiedler, 2021; Audretsch & Moog, 2022). Freedom is generally seen as positive, in that freer contexts impose less interference into the judgment of opportunities and actions, which ultimately empowers entrepreneurs. For Foucault, freedom is a condition of power, since a lack of freedom leads to the determination, and not the influencing, of the actions of others by the power holder. Freedom gives rise to power struggle and recalcitrance. Power helps advance our understanding of how freedom in a context affects entrepreneurship. It can shed light on key academic and practical questions, such as why entrepreneurs with superior technology may fail; who has the right to be an entrepreneur; what strategies entrepreneurs can use to defy powerful actors; and whether divergence from power structures by entrepreneurs is punished, tolerated, supported, or celebrated and rewarded. Power draws our attention to critical forces shaping opportunities when actors are free. Without that attention to one of the most ubiquitous and fundamental forces shaping society and the economy, power, the scholarly literature on entrepreneurship will remain limited and incomplete.

## Footnotes

1 It is the nature of entrepreneurial firms that decision and decision rights are less based on power by contracts, like in established firms, but on power based on ownership and ownership rights as well as intangible assets. Fruitful literature, reconciling the concept of power within the theory of the firm, in particular entrepreneurial firms, is by Rajan and Zingales (1998).
